# GM-CSF as a therapeutic target in autoimmune diseases

**DOI:** 10.1186/s41232-016-0014-5

**Published:** 2016-07-05

**Authors:** Aoi Shiomi, Takashi Usui, Tsuneyo Mimori

**Affiliations:** Department of Rheumatology and Clinical Immunology, Graduate School of Medicine, Kyoto University, 54-Kawahara-cho, Shogoin, Sakyo-ku, Kyoto, 606-8507 Japan

**Keywords:** GM-CSF, IL-17, Autoimmune disease, Multiple sclerosis, Rheumatoid arthritis, Crohn’s disease, Th17 plasticity, GM-CSF target therapy

## Abstract

Granulocyte-macrophage colony-stimulating factor (GM-CSF) has been known as a hematopoietic growth factor and immune modulator. Recent studies revealed that GM-CSF also had pro-inflammatory functions and contributed to the pathogenicity of Th17 cells in the development of Th17-mediated autoimmune diseases. GM-CSF inhibition in some animal models of autoimmune diseases showed significant beneficial effects. Therefore, several agents targeting GM-CSF are being developed and are expected to be a useful strategy for the treatment of autoimmune diseases. Particularly, in clinical trials for rheumatoid arthritis (RA) patients, GM-CSF inhibition showed rapid and significant efficacy with no serious side effects. This article summarizes recent findings of GM-CSF and information of clinical trials targeting GM-CSF in autoimmune diseases.

## Background

Granulocyte-macrophage colony-stimulating factor (GM-CSF) was originally defined by its ability in vivo to generate colonies of both granulocytes and macrophages from bone marrow precursors [1]. It has also been shown to act on mature myeloid cells as pro-survival, activation, and differentiation factors [2]. Recent studies suggest that GM-CSF also has many pro-inflammatory functions and plays critical roles in the development of autoimmune and inflammatory diseases [3, 4].

## Function of GM-CSF

### Myeloid cell

GM-CSF promotes the survival and activation of macrophages, neutrophils, and eosinophils, as well as dendritic cell (DC) maturation [2]. On the other hand, GM-CSF-deficient mice have relatively normal myelopoiesis with abnormal lung histology that is indistinguishable from human pulmonary alveolar proteinosis (PAP) [5], indicating a redundant role of GM-CSF in myeloid cell development and its differentiation and critical roles in the maturation and surfactant catabolism of alveolar macrophages [6]. In addition to these functions, GM-CSF is reported to have diverse functions on mature myeloid cells, including enhancement of pro-inflammatory cytokine production [7], antigen presentation [8], induction of phagocytosis [9–11], and promotion of leukocyte chemotaxis and adhesion [12, 13].

GM-CSF can polarize macrophages into M1-like inflammatory macrophages, which produce a variety of inflammatory cytokines such as TNF, IL-6, IL-12p70, IL-23, or IL-1β, and thus promote Th1-Th17 responses [7, 14, 15]. On the other hand, the association of GM-CSF and Th2 immunity is also reported in allergic airway inflammation [16, 17].

GM-CSF positively regulates the development of dermal migratory CD103^+^CD11b^−^ and gut migratory CD103^+^CD11b^+^ DCs [18, 19] but negatively regulates the development of plasmacytoid DCs (pDCs) [20] and resident CD8^+^ DCs [19]. GM-CSF is also reported to induce the development of inflammatory monocyte-derived DCs (moDCs) in vitro [21], but its effect in vivo has not been established well. It was reported that GM-CSF transgenic mice have increased the number of moDCs [22] and GM-CSF-deficient mice with inflammatory arthritis have markedly reduced the number of moDCs [23]. On the other hand, in the other reports, GM-CSF was shown to be dispensable for the differentiation of moDCs, at least during acute infections [19, 24].

In neutrophils, GM-CSF upregulates the antimicrobial functions such as phagocytosis, reactive oxygen species (ROS) production, or expression of the integrin CD11b which increases cellular adhesion and tissue entry [12, 25].

The effect of GM-CSF on osteoclast differentiation is quite complex, for it has both enhancing and suppressive actions. Under the steady state, osteoclasts are known to differentiate from hematopoietic precursors of the monocyte/macrophage lineage in the presence of M-CSF and receptor activator of NFκB ligand (RANKL) [26]. GM-CSF induces shedding of M-CSF receptor, resulting in disruption of osteoclast differentiation [27]. On the other hand, the differentiation of osteoclast precursors generated in the presence of GM-CSF or GM-CSF plus TNFα was not inhibited by GM-CSF in vitro, indicating that a different set of osteoclast precursors is available in inflammatory arthritis and that they respond to a variety of pro-inflammatory cytokines which compensate for the loss of M-CSF signaling [28, 29]. GM-CSF is also reported to induce fusion of prefusion osteoclasts to form the bone-resorbing osteoclasts and induce bone erosion [30]. Conversely, other report suggested that GM-CSF inhibited the resorption ability of osteoclasts, indicating the existence of another osteoclastic pathway [28].

### B cell

Among B cells, innate response activator (IRA) B cells, a B1a B cell-derived inflammatory subset, produce GM-CSF and also express GM-CSF receptors [31, 32]. GM-CSF controls IgM production from IRA B cells in an autocrine manner which is essential to protect from bacterial infection [31, 32].

### Neuron

Sensory nerves express GM-CSF receptors, and GM-CSF is reported as a key mediator in bone-cancer pain [33], osteoarthritis pain, and inflammatory arthritic pain [34, 35]. A sensory nerve-specific knockdown of GM-CSF receptors attenuated tumor-evoked pain [33]. GM-CSF deficiency or neutralization also abolished osteoarthritis pain and inflammatory arthritic pain [34, 35].

## GM-CSF receptor

GM-CSF receptor consists of an α-subunit which binds GM-CSF with low affinity (GMRα) and a signal-transducing βc-subunit which is shared with the IL-3 and IL-5 receptors [36]. The binary complex of GM-CSF and GMRα interacts with a free βc-subunit and forms the high-affinity hexamer complex [37]. Dodecamer complexes formed by lateral aggregation of two hexamer complexes enable Jak2 associated with a βc-subunit to dimerize and transphosphorylate, but the hexamer complexes do not [38]. This structure leads to dose-dependent responses of GM-CSF receptor activation. Low concentration of GM-CSF, as in normal condition, causes βc Ser^585^ phosphorylation and activates 14-3-3/PI-3 kinase pathway which only leads to cell survival. Higher concentration of GM-CSF, as in inflammatory condition, turns off βc Ser^585^ phosphorylation and mediated βc Tyr^577^ phosphorylation and activation of Jak2/STAT5 pathway, Ras/mitogen-activated protein kinase pathway, and PI-3 kinase pathway, resulting in promotion of cell survival, proliferation, and activation [37].

The membrane-bound GM-CSF receptor is expressed on myeloid cells [39] and on some non-myeloid cells, such as epithelial cells [40], endothelial cells [41], and neurons [33]. There also exists a soluble GM-CSF receptor alpha subunit [42]. The function of this soluble GM-CSF receptor is unclear, but it may be required to inhibit ligand binding to cells which express membrane-bound GM-CSF receptors [43].

## Production of GM-CSF

A wide variety of cells can produce GM-CSF. Major sources of GM-CSF are T and B cells, monocyte/macrophage endothelial cells, and fibroblasts. Neutrophils, eosinophils, epithelial cells, mesothelial cells, Paneth cells, chondrocytes, and tumor cells can also produce GM-CSF [44]. The production of GM-CSF is stimulated by various factors, including TNF, IL-1, toll-like receptor agonists, and prostaglandin E2 [45, 46]. Recently, the pathogenicity of GM-CSF-producing CD4 T cells in autoimmune and inflammatory diseases is clarified and gaining increasing attention [3, 4].

Recently, Th17 cells were clarified to have high plasticity [47]. The “classical” Th17 cells driven by transforming growth factor-β1 (TGFβ1) and IL-6 have been reported to be weak inducers of inflammation [48, 49]. Conversely, IL-23 together with IL-1β induces the differentiation of highly pathogenic Th17 cells (Th1/17 cells) which also express CXCR3 and T-bet and produce IL-17, IFN-γ, and GM-CSF in mice [48, 49]. Recent studies clarified the production of GM-CSF is critical for the pro-inflammatory function of Th17 cells [3, 4]. In humans, IL-12, instead of IL-23, together with IL-1β is reported to promote the differentiation of Th1/17 cells [50]. Th1/17 cells can be distinguished from Th1 cells by the expression of CD161, a hallmark of Th17 progeny cells in humans [51]. A recent study reported that IL-23 drives switch of surface signature from CCR6 to CCR2 which defines GM-CSF/IFNγ-producing inflammatory Th17 cells and that CCR2 drives these cells to the central nervous system (CNS) in experimental autoimmune encephalomyelitis (EAE) [52]. The pathway to induce GM-CSF production in Th cells has not been clarified well yet. T-bet was reported to drive CCR6^−^CCR2^+^ GM-CSF/IFNγ-producing Th17 cell formation [52]. On the other hand, T-bet-deficient Th17 cells are reported to have normal GM-CSF production [3]. Ectopic RORγt expression showed that RORγt drove GM-CSF production in Th cells [4]. Conversely, RORγt-deficient CD4 T cells were also able to produce GM-CSF [3]. These reports indicate the existence of additional pathways.

GM-CSF is also reported to be produced by Th1 cells and is crucial for their encephalitogenicity [4]. It was reported that STAT4 regulated GM-CSF production in Th1 cells but not in Th17 cells [53]. On the other hand, the other report indicated that STAT4 regulated GM-CSF production in both Th1 and Th17 cells by directly binding to the Csf2 promoter [54]. Recent findings on Th17 plasticity and heterogeneity indicate that it is necessary to re-examine previous studies in this field.

In addition to these cells, recent studies reported the existence of an IL-2- or IL-7-activated STAT5-dependent novel subset of GM-CSF-producing Th cells (Th-GM) which express low or undetectable T-bet, GATA-3, or RORγt [55, 56] and that Th-GM cells were able to induce more severe EAE than Th17 or Th1 cells [55]. In humans, the CCR10^+^CCR4^+^CXCR3^−^CCR6^−^ signature was reported to define Th-GM [56]. It is possible that Th-GM cooperate with Th1/17 cells or Th1 cells to exacerbate the development of inflammation.

Th2 cells are also reported as one of the GM-CSF-producing cells [57]. A positive correlation between GATA-3^+^ cells and GM-CSF^+^ cells in the nasal mucosa of allergic rhinitis patients is reported [58]; however, the precise mechanism of GM-CSF production in Th2 cells has not been analyzed yet.

## GM-CSF in autoimmune disease

Recent evidence revealed that GM-CSF played critical roles in the development of many autoimmune diseases. GM-CSF depletion or neutralization suppresses many autoimmune disease models, including EAE [3, 4], arthritis [59–61], arthritis-related interstitial lung disease [60], nephritis [62], or psoriasis [63]. On the other hand, GM-CSF administration is reported to improve the models of myasthenia gravis [64], type 1 diabetes [65], or colitis [66].

### GM-CSF in the CNS

IL-17-producing Th17 cells have been reported as central mediators of CNS inflammation in both EAE and multiple sclerosis (MS) [67, 68]. However, recent studies reported that GM-CSF was essential for the encephalitogenicity of CD4 T cells in EAE and that IL-17 was dispensable for the development of EAE [3, 4]. The concentrations of GM-CSF and the number of GM-CSF-producing CD4 T cells in the cerebrospinal fluid were reported to be elevated in MS patients [56, 69]. GM-CSF deficiency or neutralization was reported to prevent the onset of EAE [70, 71]. In contrast, the administration of recombinant GM-CSF exacerbated EAE [70].

GM-CSF induces the proliferation and activation of microglial cells which produce highly neurotoxic substances such as ROS, nitrogen species, and glutamate [71, 72]. GM-CSF-producing CD4 T cells also induce the polarization of neurotoxic M1-like phenotype of microglia and promote the production of pro-inflammatory cytokines such as IL-1β, IL-6, and TNFα, which also contribute to myelin sheath damage [72, 73]. GM-CSF is also required for the recruitment of peripheral myeloid cells that contribute to blood-brain barrier and blood-spinal cord barrier disruption and demyelization into the CNS [74, 75]. These resident and infiltrating antigen-presenting cells (APCs) re-stimulate T cells and lead to further APC activation [76].

### GM-CSF in arthritis

In the models of arthritis, IL-17 has been reported as a main pathogenic cytokine as in EAE [77, 78]. IL-17 deficiency ameliorated collagen-induced arthritis (CIA) but did not completely inhibit it [78]. IL-17 inhibition was also reported to be an unsatisfactory method for the treatment of rheumatoid arthritis (RA) [79]. These reports indicated the existence of the other critical factors in the development of arthritis.

In RA patients, the concentration of GM-CSF in the synovial fluid and plasma was elevated [80, 81] and the administration of recombinant GM-CSF exacerbated the disease activity [82]. Bone marrow adjacent to the RA joints contains an increased number of granulocyte-macrophage progenitors, colony-forming unit granulocyte-macrophages (CFU-GM), which can differentiate into granulocytes or macrophages with GM-CSF stimulation [83] and also into osteoclasts with M-CSF and RANKL stimulation [84]. The frequency of GM-CSF-producing T helper cells in synovial fluid cells was also significantly increased compared to peripheral blood mononuclear cells (PBMCs) and correlated with erythrocyte sedimentation rate (ESR) levels in juvenile idiopathic arthritis (JIA) [85].

In mouse models of arthritis, GM-CSF deficiency or neutralization prevented the development of arthritis [59–61] and reduced the concentrations of TNF and IL-1 in joints [59]. Conversely, GM-CSF administration exacerbated arthritis [86]. In arthritis of SKG mice, GM-CSF secreted by T cells upregulated the production of pro-inflammatory cytokines such as IL-6 or IL-1β from macrophages [60, 87]. This in turn induced further differentiation and expansion of IL-17-producing and GM-CSF-producing CD4 T cells [60] and exacerbated arthritis.

#### GM-CSF in arthritis-related interstitial lung disease

SKG arthritis model develops chronic-progressive interstitial lung disease (ILD) which histologically resembles connective tissue disease-associated ILD (CTD-ILD) [60, 88]. This model was characterized with massive infiltration of Th17 cells, GM-CSF-producing CD4 T cells, and neutrophils with fibrosis in the lungs [60]. The overexpression of GM-CSF was reported to induce severe neutrophil, eosinophil, and macrophage infiltration with fibrosis in the lungs [89, 90]. GM-CSF promotes macrophages to produce IL-6 and IL-1β and enhances differentiation of IL-17A and/or GM-CSF-producing T cells and therefore infiltration of neutrophils into the lungs [60]. Neutrophils were reported to produce ROS, MMPs, neutrophil elastase, or myeloperoxidase and cause parenchymal and stromal cell injury in the lungs [91–93]. GM-CSF also stimulates macrophages to release profibrotic cytokines and induces fibrosis by direct stimulation of airway smooth muscle cells [90, 94]. GM-CSF neutralization completely blocked the development of ILD in SKG mice but IL-17A neutralization did not, indicating that GM-CSF played a more critical role than IL-17A in this ILD [60].

The contribution of GM-CSF in human ILD has not been analyzed well yet. In patients with pulmonary fibrosis, the concentration of GM-CSF in the bronchoalveolar lavage fluid (BALF) was reported to be elevated [95, 96]. A recent report also reported that serum concentration of GM-CSF was associated with ILD in patients with RA [97]. Further studies to clarify the contribution of GM-CSF in CTD-ILD are awaited.

### GM-CSF in the intestine

In the intestine, GM-CSF contributes to mucosal barrier function and resistance to bacterial translocation by promoting the recruitment and activation of myeloid cells. GM-CSF also promotes tissue repair via acceleration of epithelial cell proliferation and macrophages as effectors of wound healing [98–100].

Recent studies suggested that mucosal innate immunodeficiency caused by a variety of genetic defects contributed the susceptibility of Crohn’s disease (CD) and increased the translocation of pathogens to the bowel tissue [101]. Higher levels of GM-CSF secretion have been detected in mucosal lesions of inflammatory bowel disease (IBD) compared with normal mucosa [102, 103] and also in the colon lesions of dextran sodium (DSS)-induced colitis mice model [104]. On the other hand, in CD, the increased levels of GM-CSF autoantibodies have been reported [105]. The levels of GM-CSF autoantibodies correlated with the disease activity and inversely correlated with the neutrophil phagocytic activity in CD patients [105]. GM-CSF-deficient mice were reported to be more susceptible to acute DSS-induced colitis [106], and the severity of this colitis was largely prevented by GM-CSF administration [66, 107]. Conversely, GM-CSF neutralization was reported to ameliorate 2,4,6-trinitrobenzene sulfonic acid (TNBS)-induced colitis [108] and IL-23-driven colitis [109]. The overexpression of GM-CSF in the stomach was reported to lead to autoimmune gastritis [110]. These data indicated the possibilities that both relative shortage and excessive amount of GM-CSF could induce colitis. Further studies are also needed to clarify whether GM-CSF autoantibodies in CD patients are pathogenic or not pathogenic and produced just as a result of elevated GM-CSF.

There are some trials of GM-CSF administration for the treatment of CD patients. Initial reports indicated a high rate of clinical response and remission with minimal adverse effects [111–113]. However, a recent large randomized trial reported that it is not effective for induction of clinical remission or improvement in active CD [114]. The pathogenic mechanism of CD patients is considered to be heterogeneous. Therefore, GM-CSF administration might be effective only in some subgroups of patients.

## GM-CSF target therapy

There are several ongoing or completed clinical trials targeting GM-CSF or GM-CSF receptor (Table 1). Detailed information is available at ClinicalTrials.gov. Although GM-CSF inhibition showed rapid clinical response with no serious adverse reactions so far [115–117], there are some potential side effects which need to be monitored. The existence of GM-CSF autoantibodies or the mutations of GM-CSF receptor are reported to cause PAP [6]. On the other hand, healthy individuals also have GM-CSF autoantibodies [118], suggesting that the risk of PAP is increased only when GM-CSF autoantibody levels are increased above a critical threshold [119]. In addition, GM-CSF inhibition might exacerbate the existing Crohn’s disease as mentioned above. An increased susceptibility to infections in GM-CSF-deficient mice [5, 120] also indicates the risk of infection in GM-CSF target therapy.

**Table 1:** Clinical trials targeting GM-CSF

Target	Drug	Indication	Trial	Phase	Regimen	Status	
GM-CSFR	Mavrilimumab (CAM-3001)	RA	NCT00771420	I	MTX + 0.01, 0.03, 0.1, 0.3, 1.0, 3.0, and 10.0 mg/kg or placebo, single dose	Completed	[121]
		RA	NCT01050998 (EARTH study)	IIa	MTX + 10, 30, 50, and 100 mg or placebo biweekly for 12 weeks	Completed	[117]
		RA	NCT01706926 (EARTH EXPLORER 1)	IIb	MTX + 30, 100, and 150 mg or placebo biweekly for 24 weeks	Completed	[122–125]
		RA	NCT01712399	IIb	Long time safety study (5 years) MTX + 100 mg biweekly	Active, not recruiting	[126, 127]
		RA	NCT01715896 (EARTH EXPLORER 2)	II	MTX + mavrilimumab biweekly or golimumab alternating with placebo	Completed	[128]
GM-CSF	MOR103	RA	NCT01023256	Ib/IIa	0.3, 1.0, and 1.5 mg/kg or placebo weekly for 4 weeks	Completed	[116]
		MS	NCT01517282	Ib	0.5, 1.0, and 2.0 mg/kg or placebo biweekly for 10 weeks	Completed	[115]
GM-CSF	Namilumab (MT203)	RA	NCT01317797	Ib	150 and 300 mg or placebo biweekly, 3 times	Completed	[129]
		RA	NCT02393378	II	MTX + namilumab or adalimumab for 24 weeks	Recruiting	[131]
		RA	NCT02379091	II	MTX + 20, 80, and 150 mg or placebo for 24 weeks	Recruiting	[130]
		Psoriasis	NCT02129777	II	40, 100, 160, and 300 mg or placebo on day 1; 20, 50, 80, and 150 mg or placebo on days 15, 43, and 71 (followed by open-label extension study)	Recruiting	[132]
GM-CSF	KB003	RA	NCT00995449	II	600 mg or placebo at weeks 0, 2, 4, 8, and 12	Terminated	[133]
		Asthma	NCT01603277	II	400 mg or placebo	Completed	
GM-CSF	MORAb-022	RA	NCT01357759	I	Escalating doses of MORAb-022 or placebo	Completed	[134]

*Abbreviations*: *GM-CSFR* GM-CSF receptor, *RA* rheumatoid arthritis, *MS* multiple sclerosis, *MTX* methotrexate

### Mavrilimumab

Mavrilimumab is a human monoclonal antibody against GM-CSF receptor α. In the first phase 1 study, 32 subjects with mild RA received single intravenous escalating doses of mavrilimumab and showed its safety and tolerability. Reductions of acute-phase reactants and disease activity score (DAS) 28 was also observed [121].

A phase 2a randomized, double-blind, placebo-controlled, ascending-dose study in subjects with moderate to severe active RA (EARTH study) reported significant efficacy with no serious adverse events [117]. In this study, 239 patients with active RA despite methotrexate (MTX) treatment received subcutaneous mavrilimumab or placebo every other week for 12 weeks on stable-background MTX therapy and 55.7 % of all mavrilimumab-treated participants met the primary end point of achieving a ≥1.2 decrease from baseline in the DAS (DAS28-CRP) vs 34.7 % of placebo-treated participants at week 12. All mavrilimumab-treated patients showed a response by week 2. The 100 mg dose of mavrilimumab demonstrated a significant effect vs placebo on DAS28-CRP <2.6, all categories of the American College of Rheumatology (ACR) criteria, and the Health Assessment Questionnaire Disability Index.

In a subsequent phase 2b study (EARTH EXPLORER 1) [122–125], 326 patients with moderate to severe RA received an ascending dose of mavrilimumab or placebo every 2 weeks plus MTX for 24 weeks and showed an acceptable safety and tolerability. A statistically significant difference in DAS28-CRP was observed in all doses of mavrilimumab vs placebo at week 12, and a significantly higher ACR response rate of mavrilimumab-treated subjects than that of placebo was observed at week 24. Particularly, the 150 mg dose showed a significant difference vs placebo for these parameters as early as week 1.

A nonrandomized, open-label phase 2 study to evaluate the long-term safety and tolerability from day 1 through to approximately 5 years is ongoing (NCT01712399) [126]. This study enrolled RA patients who had completed the EARTH EXPLORER 1 and 2 studies or were rescued as inadequate responders at a predefined time point, and they received 100 mg of mavrilimumab every other week. At week 74, mavrilimumab demonstrated sustained safety and efficacy with DAS28-CRP <3.2 and <2.5 rates of 57.3 and 38.5 %, respectively, and 68 % of patients showed no radiographic progression [127].

A randomized, double-blind, placebo-controlled phase 2 study (EARTH EXPLORER 2) to compare the safety and efficacy of mavrilimumab with those of golimumab, an anti-TNF antibody in 120 patients with moderate to severe RA who had an inadequate response to one or two anti-TNF agents, was completed [128].

### MOR103

MOR103, which is a fully human monoclonal antibody against GM-CSF, has shown preliminary evidence of safety and rapid efficacy (within 2 weeks) in a randomized, double-blind, placebo-controlled, dose-escalating phase 1b/2a trial for patients with moderate RA (*n* = 96) [116]. Patients received four times of weekly intravenous MOR103 or placebo, and subjects receiving higher doses of MOR103 (1.0 and 1.5 mg/kg) showed significant improvement in DAS28 scores and joint counts and significantly higher European League Against Rheumatism response rates than subjects receiving placebo.

MOR103 was also tested in a randomized, double-blind, placebo-controlled phase 1b trial for patients with relapsing-remitting MS or secondary progressive MS. Patients received placebo or an escalating dose of MOR103 every 2 weeks for 10 weeks and showed acceptable tolerability of MOR103 [115].

### Namilumab (MT203)

Namilumab is a human monoclonal antibody against GM-CSF. In a randomized, double-blind, dose-escalating phase 1b study, mild to moderate RA patients received three times of every 2-week injection of namilumab and showed its safety and tolerability [129]. The other trials testing namilumab is ongoing: a dose-finding phase 2 study of namilumab in combination with MTX in moderate to severe RA patients with inadequate response to MTX or one TNF inhibitor [130] and a phase 2 trial to evaluate the efficacy and safety of the combination of the existing MTX and namilumab vs adalimumab, an anti-TNF antibody in patients with moderate to severe early RA inadequately responding to MTX [131].

It is also being tested in a randomized double-blind phase 2 trial for moderate to severe plaque psoriasis [132].

### KB003

KB003 is a humanized monoclonal antibody targeting GM-CSF. A randomized phase 2 study in RA patients showed safety and tolerability in 3 months of repeated dosing [133].

### MORAb-002

MORAb-002 is a human monoclonal antibody against GM-CSF. A randomized, double-blind phase 1 trial in RA was completed recently [134].

## Conclusions

Recent studies clarified the pivotal roles of GM-CSF in the development of many autoimmune diseases. Much attention has been focused on the inhibition of GM-CSF as an attractive approach for the treatment of these diseases. Further studies to clarify the molecular mechanism of GM-CSF production and precise role of GM-CSF in the development of autoimmune disease are awaited with interest.

## Abbreviations

APC: antigen-presenting cell
CIA: collagen-induced arthritis
CTD-ILD: connective tissue disease-associated interstitial lung disease
DAS: disease activity score
DC: dendritic cell
EAE: experimental autoimmune encephalomyelitis
GM-CSF: granulocyte-macrophage colony-stimulating factor
ILD: interstitial lung disease
MS: multiple sclerosis
MTX: methotrexate
PAP: pulmonary alveolar proteinosis
RA: rheumatoid arthritis
